# Combination of AZD3463 and DZNep Prevents Bone Metastasis of Breast Cancer by Suppressing Akt Signaling

**DOI:** 10.3389/fphar.2021.652071

**Published:** 2021-05-28

**Authors:** Wenxin He, Xiankun Cao, Kewei Rong, Xiaojun Chen, Shuai Han, An Qin

**Affiliations:** ^1^ Shanghai Key Laboratory of Orthopaedic Implants, Department of Orthopaedics, Shanghai Ninth People’s Hospital, Shanghai Jiao Tong University School of Medicine, Shanghai, China; ^2^ Department of Plastic and Reconstructive Surgery, Shanghai Ninth People’s Hospital, Shanghai Jiao Tong University School of Medicine, Shanghai, China; ^3^ Guangxi Key Laboratory of Regenerative Medicine, Guangxi Medical University, Nanning, China

**Keywords:** breast cancer, bone metastasis, osteoclast, AZD3463, DZNeP, Akt

## Abstract

Osteolysis resulting from osteoclast overactivation is one of the severe complications of breast cancer metastasis to the bone. Previous studies reported that the anti-cancer agent DZNep induces cancer cell apoptosis by activating Akt signaling. However, the effect of DZNep on breast cancer bone metastasis is unknown. We previously found that DZNep enhances osteoclast differentiation by activating Akt. Therefore, we explored the use of the anti-cancer agent AZD3463 (an Akt inhibitor) along with DZNep, as AZD3463 can act as an anti-cancer agent and can also potentially ameliorate bone erosion. We evaluated osteoclast and breast cancer cell phenotypes and Akt signaling *in vitro* by treating cells with DZNep and AZD3463. Furthermore, we developed a breast cancer bone metastasis animal model in mouse tibiae to further determine their combined effects *in vivo*. Treatment of osteoclast precursor cells with DZNep alone increased osteoclast differentiation, bone resorption, and expression of osteoclast-specific genes. These effects were ameliorated by AZD3463. The combination of DZNep and AZD3463 inhibited breast cancer cell proliferation, colony formation, migration, and invasion. Finally, intraperitoneal injection of DZNep and AZD3463 ameliorated tumor progression and protected against bone loss. In summary, DZNep combined with AZD3463 prevented skeletal complications and inhibited breast cancer progression by suppressing Akt signaling.

## Introduction

As a typical highly metastatic cancer, breast cancer secretes osteolytic factors, which drive osteoclast formation. Metastasis necessitates long-term treatment and reduces survival time. Bone-targeting agents, such as bisphosphonates and denosumab, were developed to decrease the incidence of skeletal-related events. However, adverse effects, such as renal impairment, osteonecrosis of the jaw, and atypical femoral fractures, are associated with long-term treatment with these agents (Coleman et al., 2020). In addition to the abovementioned conventional agents, numerous emerging bone resorption inhibitors have been tested in early-stage clinical trials (Sousa and Clézardin, 2018; Coleman et al., 2020). The targets of these agents are diverse, including mTOR (Hadji et al., 2013; Hortobagyi, 2015), cathepsin K (Jensen et al., 2010; Liang et al., 2019), Src tyrosine kinase (Uehara et al., 2019), and the receptor tyrosine kinases VEGFR2 and MET (Lee and Smith, 2013; Smith et al., 2013; Smith et al., 2016; Escudier et al., 2018).

As a potential anti-tumor agent, DZNep downregulates PRC2 proteins (EZH2, SUZ12, and EED), and inhibits H3K27me3 methylation, thus ameliorating PRC2-mediated transcriptional repression. The PRC2 protein EZH2 interacts with Akt-1 in various cancers (Kleer et al., 2003; Piunti and Pasini, 2011; Chang and Hung, 2012; Yoo and Hennighausen, 2012; Gall Trošelj et al., 2016), and it mediates Akt activation via multiple mechanisms. First, EZH2 upregulates the PI3K subunits Pik3r1 and Pik3r3 to activate the PI3K-Akt pathway (Zheng et al., 2018). Second, EZH2 can induce Akt-1 (Ser473) phosphorylation (Yoo and Hennighausen, 2012).

DZNep also upregulates FBXO32 to activate FBXO32-mediated apoptosis of breast cancer cells (Tan et al., 2007). Notably, Akt activation induces phosphorylation of the FOXO family transcription factors and prevents them from translocating to the nucleus, ultimately inhibiting their transcription activation functions (Brunet et al., 1999; Kops et al., 1999; Stitt et al., 2004). Further, EZH2 also downregulates FOXC1, which is highly expressed in triple-negative breast cancer. This expands the potential clinical applications of EZH2-targeting therapeutic agents (Zheng et al., 2020).

Interestingly, we observed that DZNep enhances osteoclast differentiation and induces Akt-1 phosphorylation (Supplementary Figures S1A,B,D). Studies have demonstrated the critical role of the PI3K-Akt signaling axis in RANKL-induced osteoclastogenesis (Sugatani and Hruska, 2005; Moon et al., 2012). Thus, although DZNep can suppress breast cancer, it simultaneously increases osteolysis. To prevent osteoclastic resorption, we introduced another anti-cancer agent, AZD3463, that promisingly induced apoptosis by inhibiting the PI3K-Akt pathway in breast cancer (Hu et al., 2020; Ozates et al., 2021), neuroblastoma (Wang et al., 2016), glioblastoma (Asik et al., 2020; Goker Bagca et al., 2020), acute myeloid leukemia (Moharram et al., 2019), and Ewing sarcoma (Sampson et al., 2015). Akt activation is associated with worse outcomes in endocrine-related breast cancer (Pérez-Tenorio et al., 2002), so targeting this signaling pathway is a classic research focus (Guerrero-Zotano et al., 2016; Costa et al., 2018).

To our knowledge, there is a research gap regarding the use of the combination of AZD3463 and DZNep in relation to osteoclastogenesis. In this study, we demonstrated that the combination of these two agents slowed the progression of breast cancer metastasis to bone. Additionally, AZD3463 attenuated DZNep-induced bone resorption by inhibiting Akt signaling.

## Methods

### Cell Counting Kit (CCK)-8 Assays of Cell Proliferation

Primary bone marrow-derived cells were extracted from the bone marrow of the hindlimbs of 6- to 8-week-old C57BL/6J mice. After culturing in complete α-Minimum Essential Medium (MEM; containing 10% fetal bovine serum [FBS] and 1% penicillin/streptomycin) in the presence of 50 ng/ml macrophage colony-stimulating factor (M-CSF; R&D Systems, MN, United States) for 4 days, all attached cells were treated as osteoclast precursor cells (bone marrow-derived monocytes/macrophages [BMMs]), which were maintained and used for the following experiments. BMMs were subsequently induced to differentiate, using M-CSF (50 ng/ml) and RANKL (100 ng/ml, R&D Systems, MN, United States) for 5 days.

MDA-MB-231 and 4T1 breast cancer cell lines were cultured in complete high-glucose Dulbecco’s Modified Eagle Medium (DMEM) containing 10% FBS and 1% penicillin/streptomycin. All cells were maintained in a 37°C incubator containing 5% CO_2_.

CCK-8 assays (Dojindo, Kumamoto, Japan) were used to examine the proliferation of MDA-MB-231 cells, 4T1 cells, and BMMs. Cells were seeded in 96-well plates at a density of 5  ×  10^3^ cells/well (for the 24- and 48 h tests) or 1 ×  10^3^ cells/well (for the 120 h tests) and cultured at 37°C with 5% CO_2_ for 12 h to allow the cells to adhere. Next, the media were replaced with media containing the indicated concentration of agents until the indicated time points. Thereafter, the medium was replaced with 10% (v/v) CCK-8 in either complete high-glucose DMEM or α-MEM, and the cells were incubated for an additional 2 h. An Infinite M200Pro microplate reader (Tecan Trading AG, Hombrechtikon, Switzerland) was used to measure the absorbance at 450 nm. Each experiment was conducted in triplicate.

### Osteoclast Differentiation Assays, Bone Resorption Assays, RNA Extraction, and RT-qPCR Analysis

For osteoclast differentiation assays, M-CSF-dependent BMMs were seeded in 96-well plates at a density of 1 × 10^4^ cells/well in complete α-MEM and stimulated with 100 ng/ml RANKL, as well as with indicated doses of AZD3463 and/or DZNep. The media were changed every 2 days. After 5–6 days of incubation, when large multinucleated osteoclasts were observed in the RANKL-only positive control group, the cells were fixed with 4% paraformaldehyde (PFA) and stained to detect the osteoclastic turnover marker tartrate-resistant acid phosphatase (TRAP).

For bone resorption assays, M-CSF-dependent BMMs were seeded in hydroxyapatite-coated Osteo Assay Surface Polystyrene Microplates (Corning Inc., NY, United States) at a density of 1.3 × 10^4^ cells/well in complete α-MEM and stimulated with 100 ng/ml RANKL and respective agents for 5 days. Thereafter, the wells were incubated in 5% sodium hypochlorite solution to remove the cells. The bone resorption pits (stained using Von Kossa stain) on the microplates were captured using a phase-contrast inverted light microscope (IX71; Olympus, Hamburg, Germany).

Total RNA was extracted from BMMs stimulated with RANKL in the presence or absence of AZD3463 and/or DZNep for 5 days, using an RNA miniprep kit (Axygen, AZ, United States) according to the manufacturer’s protocol. Thereafter, cDNA was synthesized from the total RNA using a PrimeScript RT Master Mix (Perfect Real Time) cDNA synthesis kit (RR036; Takara Bio Inc., Dalian, China) according to the manufacturer’s instructions. RT-qPCR was performed on an ABI Flex 6 Real-Time PCR System (Applied Biosystems, CA, United States) using TB Green® Premix Ex Taq™ (Tli RNaseH Plus) (RR420; Takara Bio Inc.). Each reaction was performed in triplicate. Primer sequences of osteoclastogenesis-related genes are listed in Table 1. Relative fold changes in gene expression were calculated using the comparative CT (2^−ΔΔCT^) method.

**TABLE 1:** Osteoclastogenesis-related gene primer sequences.

Gene	Forward primer sequence	Reverse primer sequence
*Ctsk*	TAG​CCA​CGC​TTC​CTA​TCC​GA	CCT​CCG​GAG​ACA​GAG​CAA​AG
*Calcr*	TCT​GCG​TTC​CTG​AGA​ACA​CC	AAG​GCG​CTC​TAA​TGG​CAC​TT
*Nfatc1*	CTT​CGA​GTT​CGA​TCA​GAG​CGG	AGG​GTC​GAG​GTG​ACA​CTA​GG
*Acp5*	CAC​TCC​CAC​CCT​GAG​ATT​TGT	CAT​CGT​CTG​CAC​GGT​TCT​G
*Atp6v0d2*	GCA​GAG​CTG​TAC​TTC​AAT​GTG​G	TAG​TCC​GTG​GTC​TGG​AGA​TG
*Fos*	TGT​TCC​TGG​CAA​TAG​CGT​GT	TCA​GAC​CAC​CTC​GAC​AAT​GC
*Mmp9*	CCC​TGG​AAC​TCA​CAC​GAC​AT	TGG​TTC​ACC​TCA​TGG​TCC​AC
*Actb*	ACA​GCA​GTT​GGT​TGG​AGC​AA	ACG​CGA​CCA​TCC​TCC​TCT​TA
*Gapdh*	ACC​CAG​AAG​ACT​GTG​GAT​GG	CAC​ATT​GGG​GGT​AGG​AAC​AC

### Breast Cancer Cell Colony Formation, Migration, and Invasion Assays

For colony formation assays, MDA-MB-231 or 4T1 cells, at a density of 1 × 10^3^ cells/well, were evenly seeded in six-well plates. The cells were treated with AZD3463 and/or DZNep and left to grow for 5 days until colonies were visible. After washing with 1X phosphate-buffered saline (PBS), the visible colonies were fixed with methanol and stained with 0.1% crystal violet (Sigma-Aldrich, Saint Louis, MO, United States). The whole field of view of each well was then scanned using an Epson Perfection V600 Photo Scanner (Seiko Epson Corp., Nagano, Japan), and the total area taken up by colonies was assessed using Fiji 1.0 (NIH, United States).

For wound healing assays, MDA-MB-231 or 4T1 cells were seeded in six-well plates at a density of 5  ×  10^5^ cells/well. After reaching 100% confluency, a scratch was produced in the center of each well using a sterile 1-mL pipette tip vertically. PBS was used to rinse off the detached cells, followed by incubation with or without AZD3463 and DZNep (at the indicated concentrations) in serum-free medium for an additional 24 or 48 h. Images were acquired at 0, 24, and 48 h using the IX71 inverted light microscope. Widths of the wounds were then measured, representing migration capacity.

For transwell migration assays, 5  ×  10^4^ of MBA-MB-231 or 4T1 cells were seeded in upper transwell chambers. After 12 h, the migrated cells were fixed with 4% PFA, rinsed three times with PBS, stained with 0.1% crystal violet (Sigma-Aldrich) for 10  min, and rinsed three times with PBS. Five random fields of view were photographed and the percentage of the area covered by cells was assessed.

For transwell invasion assays, Matrigel (356234; Corning Inc., NY, United States) was coated onto the upper surface of polycarbonate filters. Thereafter, 5 × 10^4^ of MBA-MB-231 or 4T1 cells were seeded onto the surface of the pre-set Matrigel in transwell upper chambers. After 24 h, the invasive cells were fixed with 4% PFA, rinsed three times with PBS, stained with 0.1% crystal violet (Sigma-Aldrich) for 10  min, and rinsed three times with PBS. Five random fields of view were photographed and the percentage of the area covered by cells was assessed.

### Western Blotting

MDA-MB-231 breast cells were cultured with AZD3463 and/or DZNep for 3 days. BMMs were pre-stimulated with AZD3463 or DZNep for 2 h in serum-free medium and then stimulated with RANKL (100 ng/ml) for the indicated time periods.

Total proteins from whole cells were extracted with radioimmunoprecipitation assay (RIPA) lysis buffer (medium-level intensity; Beyotime, Shanghai, China) in the presence of phenylmethylsulfonyl fluoride (PMSF), protease inhibitor, and phosphatase inhibitor cocktails. Subsequently, the proteins were separated using ExpressPlus polyacrylamide gel electrophoresis (PAGE) gels (GenScript Laboratories, Piscataway, NJ, United States) and transferred onto polyvinylidene fluoride (PVDF) membranes using an e-blot device (GenScript Laboratories). The membranes were blocked with 5% (w/v) skim milk in tris-buffered saline (TBS) containing 0.1% (v/v) Tween-20 (TBST, pH 7.4), and incubated for 1 h at room temperature. The membranes were then washed three times for 15 min with TBST and incubated for 12 h at 4°C with primary antibodies (Cell Signaling Technology) against the following proteins: EZH2 (#5246), p-Akt (Ser473; #4060), Akt (#9272), p-p38 (#9211), p38 (#9212), p-ERK (#9101), ERK (#9102), and Actb (#3700). The membranes were then washed three times with TBST and incubated with either an IRDye 800CW anti-mouse secondary antibody (LI-COR Biosciences, Lincoln, NE, United States) or a fluorescent anti-rabbit secondary antibody (LI-COR Biosciences), at 1:10000 dilution. Finally, the membranes were washed three times with TBST and visualized using an Odyssey near-infrared fluorescence imaging system (LI-COR Biosciences). The grayscale ratio of each phospho-protein to its respective total protein was calculated. The ratios were then standardized, using the first control lane as the baseline (1.00), as shown below each western blot lane in the figures.

### Animal Model

Female BALB/c (7 week-old) nude mice were purchased from Jihui Laboratory Animal Care Co., Ltd. (Shanghai, China) and maintained in a specific-pathogen-free animal facility. An intratibial murine model of breast cancer bone metastasis (Pang et al., 2020) was created by harvesting MDA-MB-231 cells and resuspending them in sterile PBS (1 × 10^6^ cells/ml) and then injecting 100 μl of the cell suspension into the left tibia plateau of each mouse. After being observed for 1 week, 24 mice in good condition were randomly allocated to four groups (*n* = 6 per group) and then intraperitoneally injected twice per week with PBS, AZD3463 (7.5 mg/kg body weight), DZNep (2 mg/kg body weight), or AZD3463 (7.5 mg/kg body weight) plus DZNep (2 mg/kg body weight). The mice were weighed and tumor volumes (length and width, volume = length × width^2^/2) were measured every week for 5 weeks. Thereafter, the mice were sacrificed, weighed, and underwent X-rays. Left tibiae (with tumors), kidneys, and livers were fixed in 4% PFA for 24–48 h. The tibiae were analyzed by micro-computed tomography (CT) and then decalcified in 10% ethylenediaminetetraacetic acid (EDTA; pH 7.4) for 28 days. Finally, the tissues were embedded in paraffin and sliced for histological examination, including hemotoxylin and eosin (H&E) staining and immunofluorescence staining. All procedures were performed following protocols approved by the Institutional Animal Care and Use Committee of Shanghai Ninth People’s Hospital, Shanghai Jiao Tong University, School of Medicine.

### X-Ray and Micro-CT Scans

The left hindlimbs were scanned using a MultiFocus Digital Radiography System (Faxitron Bioptics LLC, Tucson, AZ, United States). The trabecular morphometry of the metaphyseal region of the proximal tibiae was quantified with micro-CT (µCT100; Scanco Medical AG, Bassersdorf, Switzerland) using an X-ray tube voltage of 70 kV and a current of 200 μA. The resolution was set to 10 μm, and 7 mm of the bone sample was acquired for each CT scan. The 3D images of the scans were reconstructed and analyzed using the µCT100 program. Trabecular bone was evaluated at 0.5 mm below the cranial growth plate and 5 mm from the caudal growth plate. Trabecular morphometry was characterized by measuring the bone volume fraction (BV/TV), trabecular thickness (Tb.Th), trabecular number (Tb.N), and trabecular spacing (Tb.Sp).

### Cancerous Tibial Bone, Kidney, and Liver Histology, and Immunofluorescence Analyses

Cancerous tibial bone, kidney, and liver sections were prepared for H&E staining, as well as immunofluorescence detection of p-Akt. For immunofluorescence detection of p-Akt, the slides were rehydrated and underwent antigen retrieval using phosphate buffer solution at pH 6.0 and 95°C for 10 min. Tissue sections were blocked to prevent non-specific binding by incubation in TBST containing 5% bovine serum albumin for 1 h. Sections were then incubated with primary antibody against p-Akt (Ser473, #4060; Cell Signaling Technology) overnight at 4°C. The next day, the tissue sections were washed and then incubated with Alexa Fluor 555-conjugated secondary antibody (Cell Signaling Technology) at 20°C for 1 h in the dark. Nuclei were counterstained with 4',6-diamidino-2-phenylindole (DAPI; Sigma-Aldrich). The sections were then washed and imaged using confocal microscopy.

### Statistical Analyses

Data were compared among multiple groups by one-way analysis of variance (ANOVA), using built-in statistical modules in GraphPad Prism 8 (GraphPad Software, San Diego, CA, United States). If a significant effect was detected by ANOVA, post-hoc Student’s *t*-tests were performed to assess pairwise differences. Data are presented as mean ± standard deviation (SD), with at least three replicates being conducted; *p* < 0.05, *p* < 0.01, and *p* < 0.001 were considered statistically significant, highly statistically significant, and extremely statistically significant.

## Results

### AZD3463 Decreased DZNep-Induced Osteoclastogenesis

**FIGURE 1:**
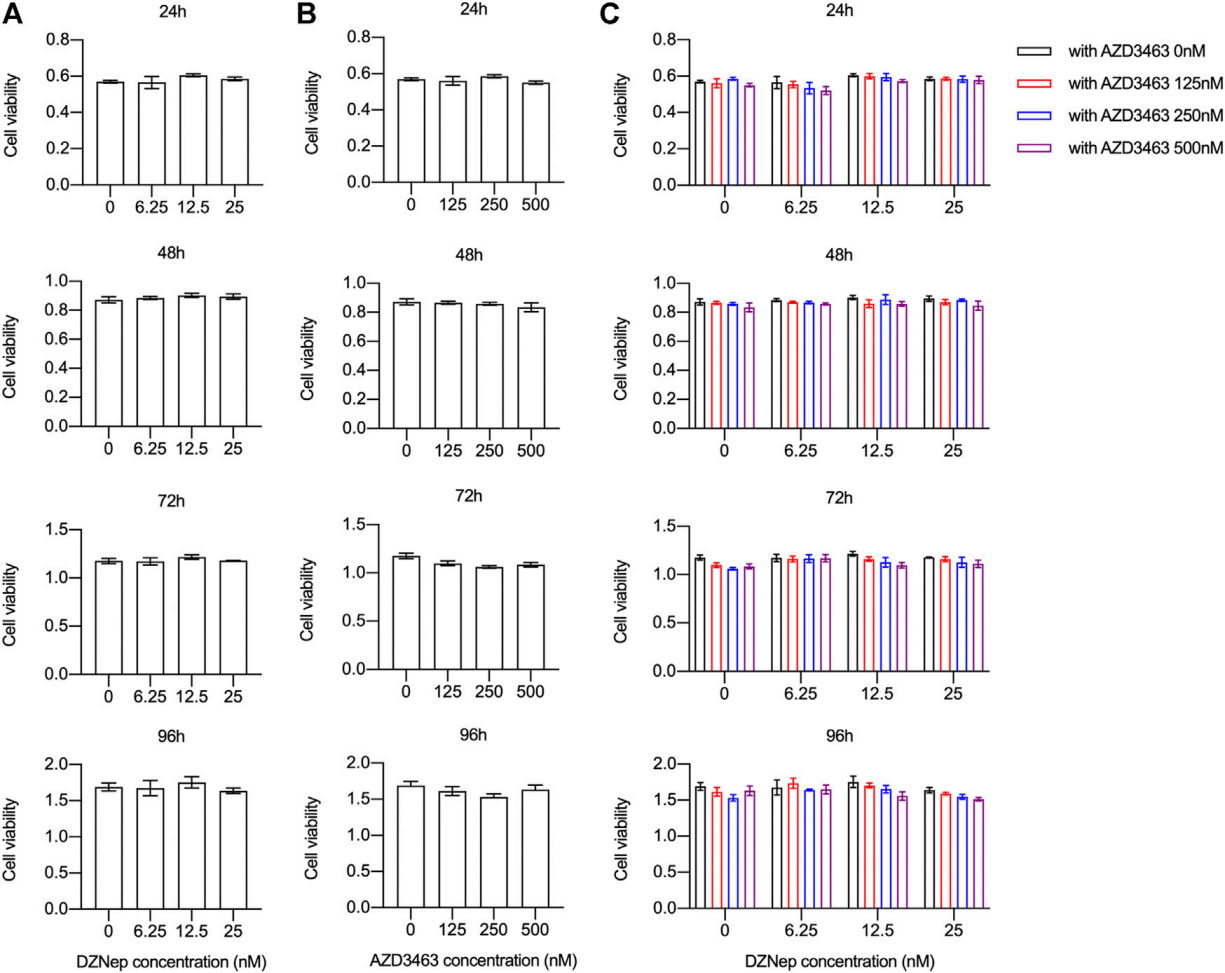
DZNep and AZD3463 did not affect BMM proliferation *in vitro*. BMM viability and proliferation remained stable after treatment with **(A)** DZNep (6.25, 12.5, and 25 nM) for 24, 48, 72, and 96 h, **(B)** AZD3463 (125, 250, and 500 nM) for 24, 48, 72, and 96 h, and **(C)** DZNep (6.25, 12, and 25 nM) plus AZD3463 (125, 250, and 500 nM) for 24, 48, 72, and 96 h.

**FIGURE 2:**
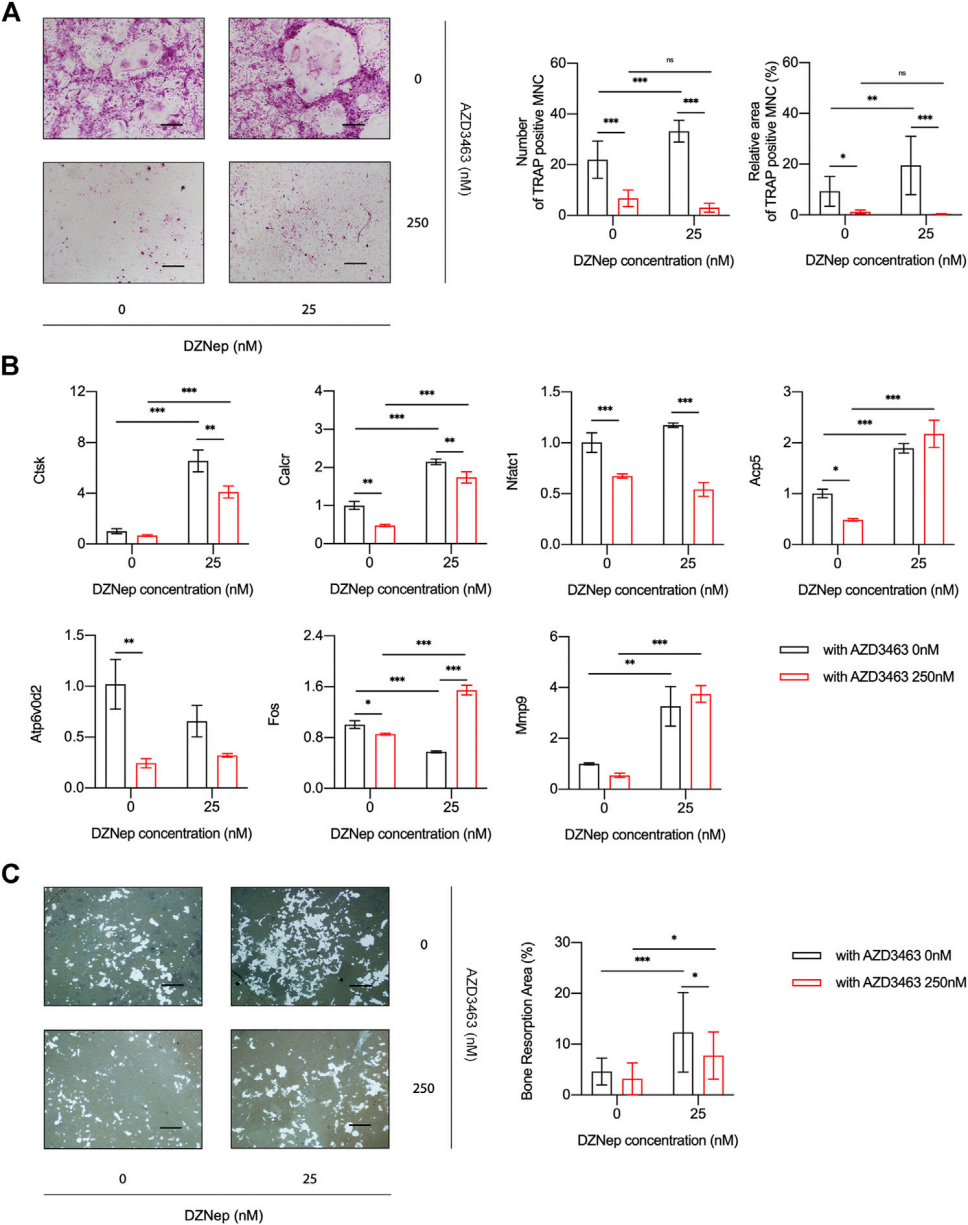
DZNep and AZD3463 influenced osteoclastogenesis *in vitro*. **(A)** TRAP staining indicated increased osteoclast differentiation after treatment with DZNep (25 nM), which was prevented by AZD3463 (250 nM). Scale bar = 10 μm. **(B)** Osteoclast differentiation-related mRNA expression increased in the DZNep (25 nM) group and decreased in the AZD3463 (250 nM) group. **(C)** Bone resorption assay using hydroxyapatite-coated microplates showed a larger area of resorption pits after treatment with DZNep (25 nM), which was alleviated by AZD3463 (250 nM). Scale bar = 10 μm. MNC: multinucleated cell.

BMM viability and proliferation after treatment with 6.25, 12.5, or 25 nM DZNep or 125, 250, or 500 nM AZD3463 were the same as the negative control levels (Figures 1A,B). Also, the combination of the two agents at the above concentrations did not cause any significant toxicity (Figure 1C). There were more and larger TRAP-positive multinucleated cells (nuclei ≥3 were counted) in the 25 nM DZNep group than the control group. BMMs failed to differentiate into osteoclasts after treatment with 250 nM AZD3463, with or without DZNep (Figure 2A). Most of the osteoclast-related genes (*Ctsk*, *Calcr*, and *Mmp9*) were upregulated in the 25 nM DZNep group and downregulated in the 250 nM AZD3463 and combination groups compared to the negative control group (Figure 2B). Similar to the TRAP staining results, the bone resorption pit area was larger in the 25 nM DZNep group compared to the negative control group. However, the addition of 250 nM AZD3463 reduced the resorption area (Figure 2C).

### AZD3463 Plus DZNep Inhibited Breast Cancer Cell Proliferation, Migration, and Invasion

**FIGURE 3:**
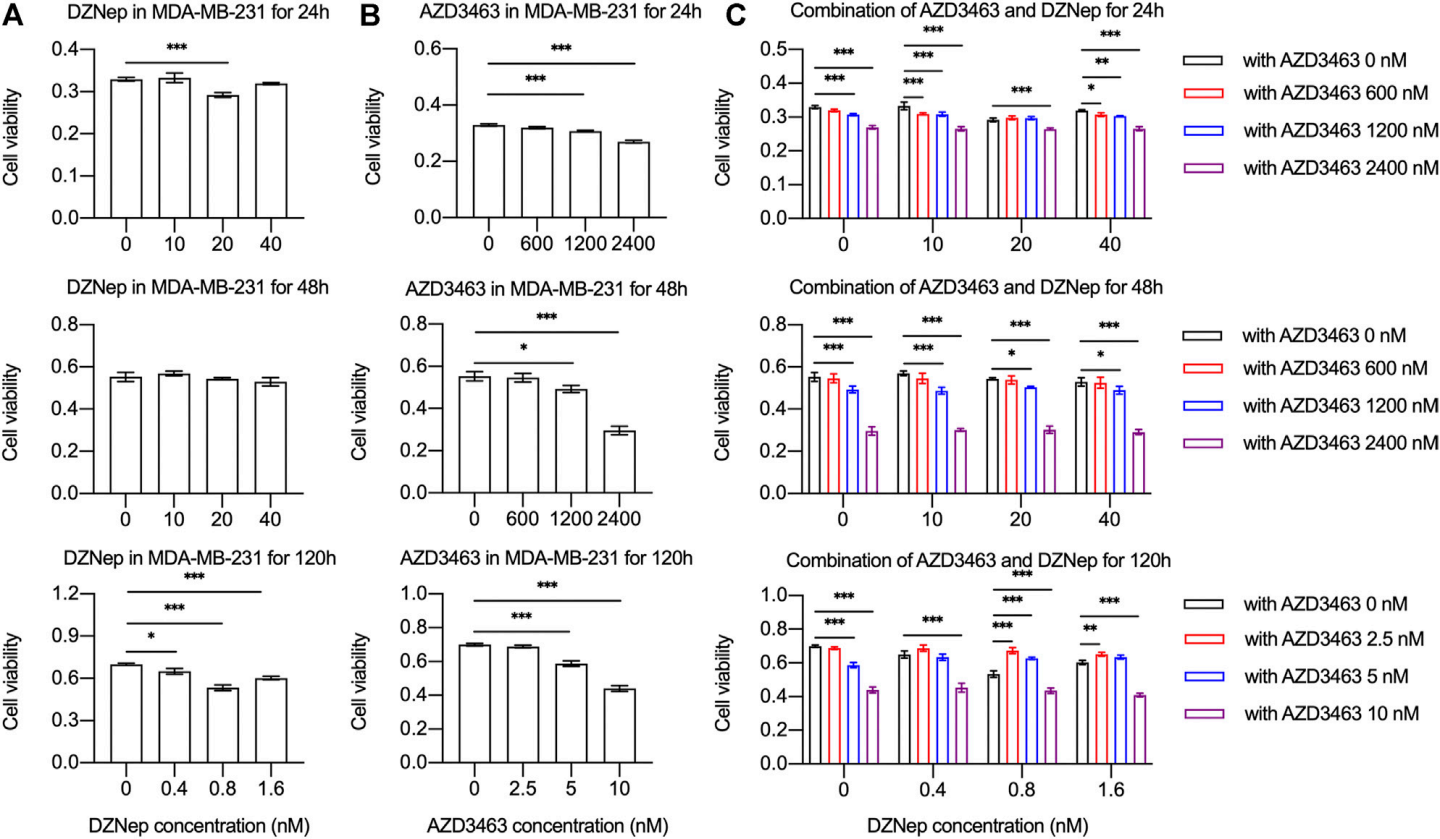
DZNep and AZD3463 inhibited MDA-MB-231 breast cancer cell proliferation *in vitro*. MDA-MB-231 cell viability and proliferation **(A)** remained stable after treatment with DZNep (10, 20, and 40 nM) for 24 and 28 h, and with DZNep (0.4, 0.8, and 1.6 nM) for 120 h, **(B)** significantly decreased after treatment with AZD3463 (1,200, and 2,400 nM) for 24 and 48 h, and with AZD3463 (5, and 10 nM) for 120 h, and **(C)** significantly decreased after treatment with AZD3463 (1,200, and 2,400 nM) and combination treatment (DZNep: 10, 20, and 40 nM; and AZD3463: 600, 1,200, and 2,400 nM) for 24 and 48 h, and after treatment with AZD3463 (5, and 10 nM) and combination treatment (DZNep: 0.4, 0.8, and 1.6 nM; and AZD3463: 2.5, 5, and 10 nM) for 120 h.

**FIGURE 4:**
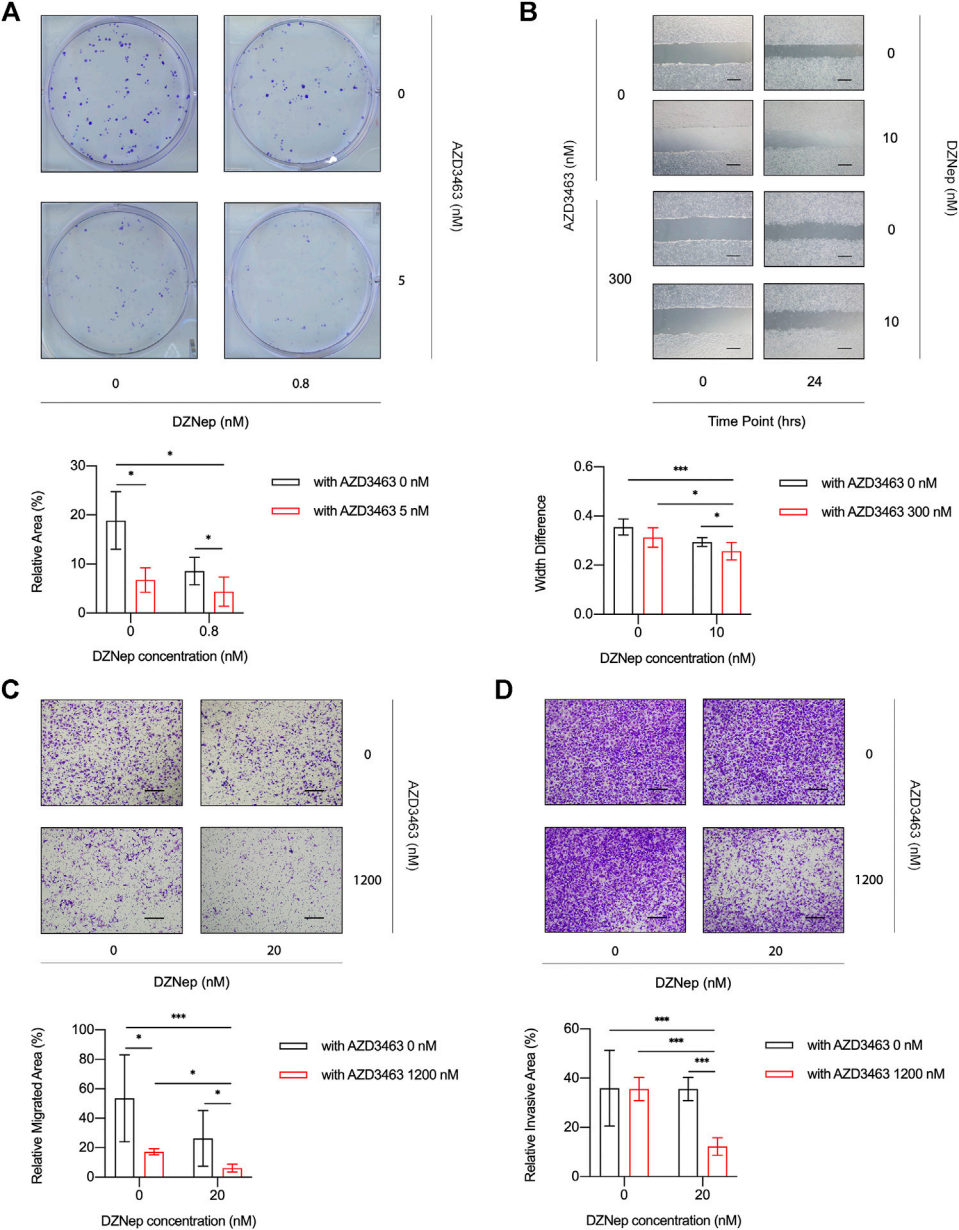
DZNep and AZD3463 inhibited breast cancer cell proliferation, migration, and invasion *in vitro*. **(A)** MDA-MB-231 cells formed fewer colonies at day 5 in the presence of AZD3463 (5 nM) or DZNep (0.8 nM), and the effect of DZNep was enhanced by AZD3463. **(B)** Wound healing assays showing that AZD3463 (300 nM) and DZNep (10 nM) inhibited scratch wound healing compared to the control group at 24 h, and the combination group had the largest gap at 24 h. Scale bar = 10 μm. **(C)** Transwell assays showing that the relative area of migrated (C) cells was decreased by AZD3463 (1,200 nM) and DZNep (20 nM), and the combination (20 nM DZNep and 1,200 nM AZD3463) group had the largest decrease. Scale bar = 10 μm. **(D)** Transwell assays showing that the relative area of invasive (D) cells decreased in the combination (20 nM DZNep and 1,200 nM AZD3463) group, while other groups were similar to the control group. Scale bar = 10 μm.

The cell viability of breast cancer cell lines MDA-MB-231 or 4T1 treated with AZD3463 (1,200 and 2,400 nM) significantly decreased compared to the control levels at 24 and 48 h (Figure 3A, Supplementary Figure S2A). There was only a tendency to decrease regarding cell viability in the DZNep (10, 20, and 40 nM) groups (Figure 3B, Supplementary Figure S2B). The combination treatment results showed that only AZD3463 (not DZNep) decreased cell viability at the concentrations used (Figure 3C, Supplementary Figure S2C). Nevertheless, relatively long-term (5 day) treatment with AZD3463 (2.5, 5, and 10 nM) decreased the proliferation of breast cancer cells (both MDA-MB-231 and 4T1), but DZNep (0.4, 0.8, and 1.6 nM) did not had a similar inhibitory effect in MDA-MB-231 cells (Figures 3A,B, Supplementary Figure S2A). Additionally, combination treatment impaired the colony formation capacity (Figure 4A, Supplementary Figure S3A).

To evaluate the migration of cancer cells, we measured scratch wound widths at 24 or 48 h. Compared to the control, AZD3463 (300 nM), and DZNep (10 nM) groups, the combination group (300 nM AZD3463 and 10 nM DZNep) had a wider gap (Figure 4B, Supplementary Figures S3A,B). Moreover, the migration in transwell assays was decreased in the combination group compared to the AZD3463 (1,200 nM) or DZNep (20 nM) groups (Figure 4C, Supplementary Figure S3C). Similarly, the combination group exhibited efficient prevention of cell movement across the Matrigel coating the transwell inserts (Figure 4D, Supplementary Figure S3D).

### Akt Signaling Played a Critical Role in the Effects of AZD3463 Plus DZNep

**FIGURE 5:**
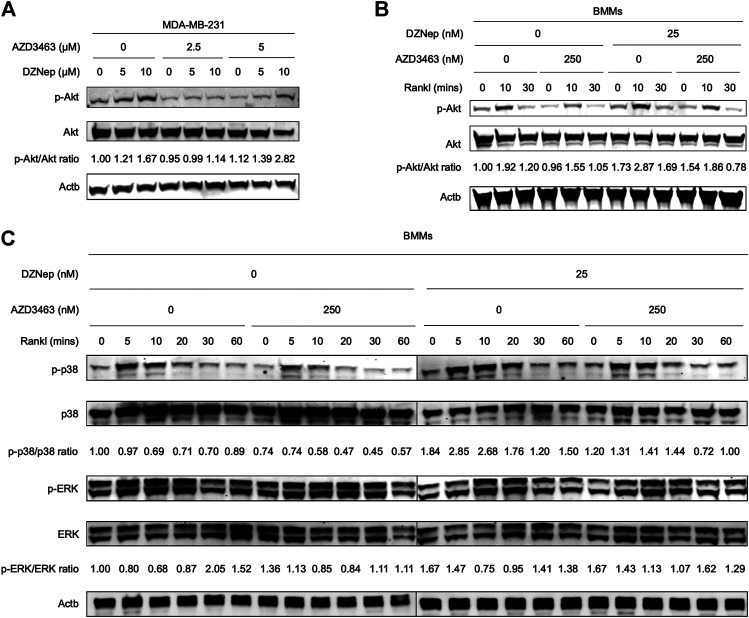
Mechanistic analyses of DZNep and AZD3463 in breast cancer cells and during RANKL-induced osteoclastogenesis. **(A)** DZNep dose-dependently upregulated p-Akt (Ser473) protein but slightly downregulated pan-Akt protein in MDA-MB-231 cells. **(B)** DZNep upregulated p-Akt (Ser473) protein, but AZD3463 ameliorated this, in BMMs during RANKL-induced osteoclastogenesis. **(C)** During RANKL-induced osteoclastogenesis, DZNep upregulated p-p38 but AZD3463 ameliorated this, and there were no significant changes in ERK signaling.

To better understand *in vitro* osteoclastic and cancerous phenotypes, we quantified the protein expression levels in cells treated with AZD3463 and/or DZNep. DZNep dose-dependently activated p-Akt (Ser473) but slightly downregulated pan-Akt protein in MDA-MB-231 cells (Figure 5A). In contrast, AZD3463 ameliorated the activation of p-Akt (Ser473). During RANKL-induced osteoclastogenesis, p-Akt (Ser473) in BMMs showed similar results to p-Akt (Ser473) in breast cancer cells (Figure 5B). We also investigated the two other essential osteoclastogenesis pathways. We found that the p38 pathway was activated by DZNep but inhibited by AZD3463, while ERK signaling appeared to be unaffected by these two agents (Figure 5C).

### AZD3463 Plus DZNep Prevented Bone Resorption and Breast Cancer Metastasis to Bone

**FIGURE 6:**
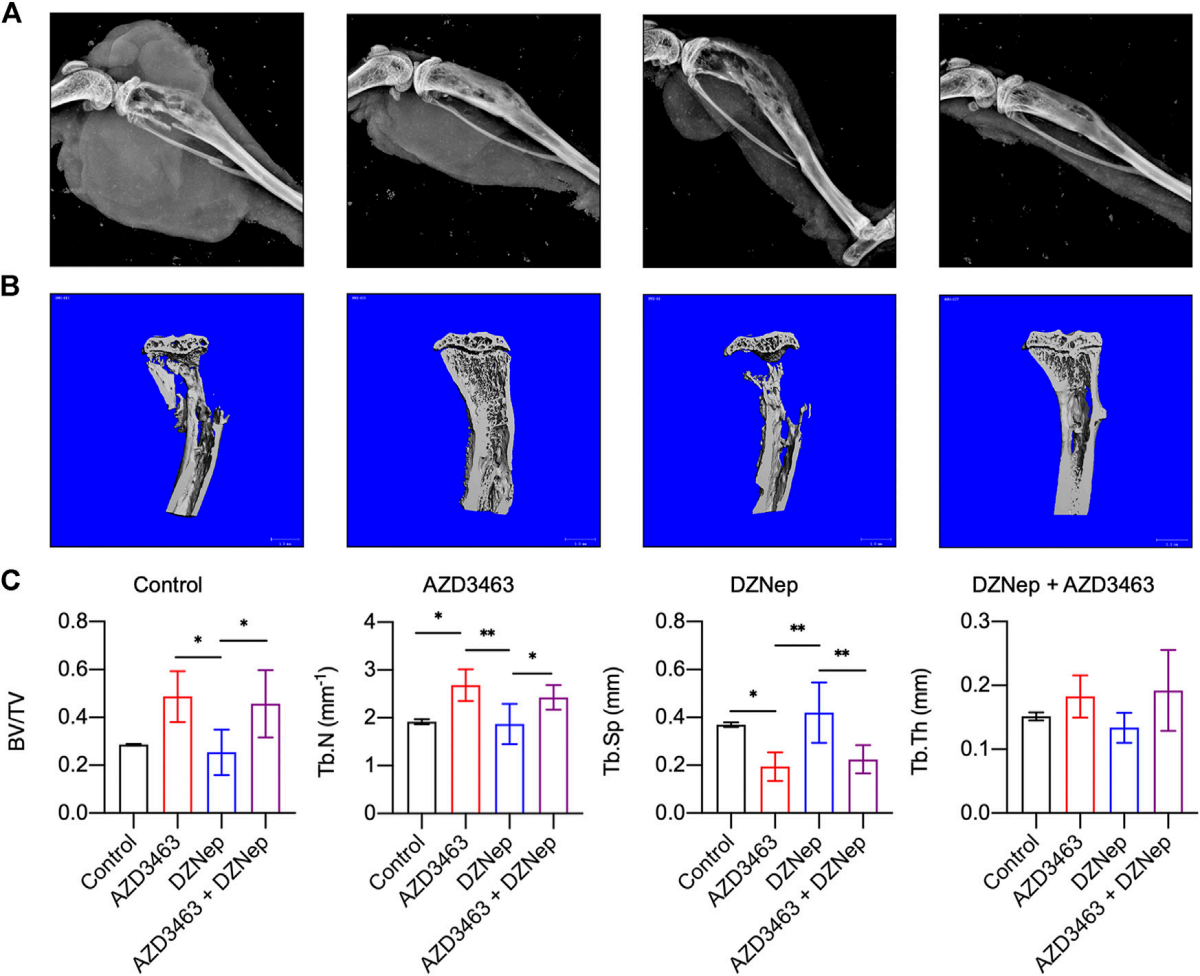
Effects of DZNep and AZD3643 on bone defects due to breast cancer metastasis *in vivo*. **(A)** X-ray images of left hindlimbs showing small tumor volumes in AZD3463 (7.5 mg/kg) and DZNep (2 mg/kg) groups, and minimum tumor volume in the combination (7.5 mg/kg AZD3463 and 2 mg/kg DZNep) group. Bone defects were less apparent in the AZD3463 and combination groups than the control and DZNep groups. **(B,C)** Micro-computed tomography (CT) indicated higher BV/TV, Tb.Th, and Tb.N and lower Tb.Sp in the AZD3463 and combination groups than the control and DZNep groups.

Reconstructed left hindlimb X-ray images showed that tumor volumes appeared to be small in the AZD3463 and DZNep groups, with those of the combination group being smallest, compared to the control group (Figure 6A). Furthermore, bone defects were less apparent in the AZD3463 and combination groups than the control and DZNep groups.

Regarding the skeletal phenotypes based on micro-CT results, BV/TV, Tb.Th, and Tb.N were decreased and Tb.Sp was increased in the DZNep group compared to the AZD3463-related groups, and the first three variables were increased, whereas the fourth variable was decreased, by adding AZD3463 compared to DZNep-only treatment (Figure 6C). This indicates that AZD3463 reduced the bone loss related to breast cancer metastasis and DZNep side effects (Figure 6C).

**FIGURE 7:**
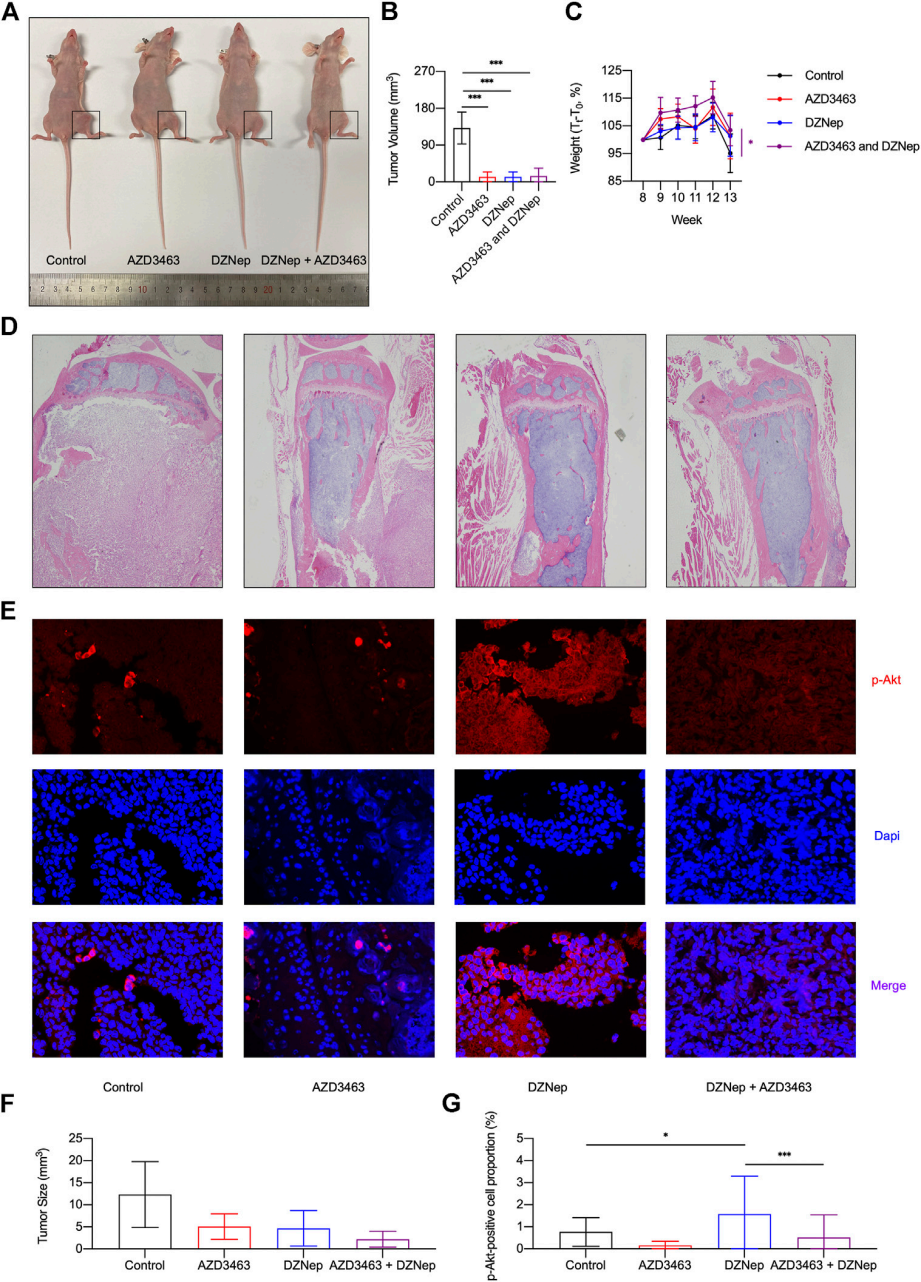
Effects of DZNep and AZD3643 on breast cancer bone metastasis *in vivo*. **(A,B)** Representative images of tumor model mice. Tumor volume of 13 week-old mice significantly decreased in AZD3463, DZNep, and combination groups compared to the control group. **(C)** Standardized body weight (T_t_/T_0_, %) of mice from week 8 (T_0_) to week 13 showing that DZNep and AZD3463 reduced weight loss. **(D,F)** Representative images and bar graph of hemotoxylin and eosin (H&E) staining of cancerous bone. **(E,G)** Representative images and bar graph of immunofluorescence (IF) staining of *p*-Akt (red), DAPI (blue), and merged results (purple).

Similar to the X-ray results (Figure 6A), H&E staining indicated that DZNep, AZD3463, and combination treatment all decreased the tumor size (Figures 7D,F). The proportion of cells that were positive for p-Akt protein was assessed by immunofluorescence staining *in vivo*, with the proportion of positive cells increasing in the DZNep group, which was ameliorated by AZD3463 (Figures 7E,G).

Similar to the X-ray results, tumor volume significantly decreased in the AZD3463, DZNep, and combination groups compared to the control group (Figures 7A,B). To some extent, the two agents significantly decreased the weight loss caused by cancer cachexia (Figure 7C). To determine whether the doses caused toxicity in mice, kidney and liver sections were assessed and there were no apparent pathological changes (Supplementary Figures S4A,B).

## Discussion

In this study, we performed *in vitro* and *in vivo* experiments using two osteoclast-targeting anti-cancer agents, DZNep and AZD3463, to evaluate their efficacy against breast cancer metastasis-induced bone loss.

AZD3463 eliminated the DZNep-induced osteoclast differentiation and bone resorption function. Inducing PI3K/Akt signaling promotes RANKL-induced osteoclastogenesis by activating the GSK3β/NFATc1 signaling cascade (Marie, 2012; Moon et al., 2012). Additionally, p-p38 signaling is required for c-Fos and NFATc1 activation in osteoclast differentiation (Li et al., 2002; Huang et al., 2006), which is consistent with our mRNA and protein expression results in BMMs. DZNep’s role as an EZH2 inhibitor should not be disregarded, though it was not examined in this study. A previous study showed that EZH2 knockdown in mice resulted in more brittle and thinner cortical bone than in wildtype mice(Hemming et al., 2017). This may be due to excessive osteoclast activation because the serum levels of CTX and TRAP (osteoclastic turnover markers) and the numbers of osteoclasts were elevated (Hemming et al., 2017). A recent study discovered that AZD3463 suppresses breast cancer metastasis to bone via modulation of the PI3K-Akt pathway (Hu et al., 2020). However, the previous study did not explore whether AZD3463 can prevent osteolysis of bone metastases by blocking osteoclastogenesis, and we were able to clarify this.

Our findings that AZD3463 inhibited breast cancer cell migration and Akt signaling were consistent with previous research (Hu et al., 2020). Activation of Akt signaling by DZNep is just the tip of the iceberg of mechanisms of DZNep. DZNep downregulates PRC2 proteins (EZH2, EED, and SUZ12), and further blocks H3K27me3 methylation (Fiskus et al., 2009; Puppe et al., 2009; Hayden et al., 2011). In particular, DZNep-induced inhibition of AdoHcy hydrolase reduces methyl group availability and thereby blocks methyltransferases (Chiang and Cantoni, 1979; Glazer et al., 1986a, 1986b; Chiang et al., 1992; Liu et al., 1992). The canonical Akt signaling pathway has been confirmed in many cells including breast cancer cells (Puppe et al., 2009; Suvà et al., 2009; Nagel et al., 2010; Choudhury et al., 2011; Hayden et al., 2011; Girard et al., 2014). Notably, DZNep did not increase the inhibitory effect of AZD3463 on cell viability but it did further inhibit colony formation. Because the agents were administered earlier in the colony formation assays (mixing agents in the medium before seeding cells) than in the cell viability assays (adding agents at 24 h after seedig cells), the results suggested that DZNep also inhibited cell attachment capacity.

To generalize the findings to other metabolic bone diseases, first, osteoblast functions should also be evaluated because both osteoclasts and osteoblasts interact in the bone remodeling cycle. EZH2-specific knockout in early mesenchymal stem cells results in multiple defects in trabecular bone formation and microarchitecture (Hemming et al., 2017). Further investigation about whether DZNep influences osteoclasts via osteoblasts would also be useful. As Akt activation is critical for increasing osteoclast formation and osteolysis in myeloma, inhibition of Akt signaling is also meaningful in this disease (Cao et al., 2013). Moreover, DZNep treatment appears to be effective in myeloma cells (Xie et al., 2011). This suggests that AZD3463 plus DZNep may achieve similar results in myeloma as in the current study. AZD3463 appears to be a promising agent to ameliorate osteoclast-related osteolysis whereas DZNep ameliorates pathological conditions involving increased bone volume. Furthermore, activation of p38 MAPK in myeloma cells promotes osteoclast maturation and bone erosion (He et al., 2012).

Although a range of treatments has been shown to prevent osteolysis during breast cancer metastasis to bone (Coleman et al., 2020), alternatives also need to be studied because of the adverse effects and individual sensitivities related to current agents. Once agents with versatile effects (simultaneous anti-bone resorption and anti-tumor effects) are discovered and their mechanisms are explored, and once genetic screening tests are available to more patients, it may be possible to select highly effective and safe targeted therapies for each patient.

## Data Availability

The original contributions presented in the study are included in the article/Supplementary Material, further inquiries can be directed to the corresponding author.

## References

[B1] Asik A. Ay N. P. O. Bagca B. G. Caglar H. O. Gunduz C. Avci C. B. (2020). Combination of Salinomycin and AZD3463 Reveals Synergistic Effect on Reducing the Viability of T98G Glioblastoma Cells. Acamc 20, 2267–2273. 10.2174/1871520620666200721121517 32698744

[B2] Brunet A. Bonni A. Zigmond M. J. Lin M. Z. Juo P. Hu L. S. (1999). Akt Promotes Cell Survival by Phosphorylating and Inhibiting a Forkhead Transcription Factor. Cell 96, 857–868. 10.1016/S0092-8674(00)80595-4 10102273

[B3] Cao H. Zhu K. Qiu L. Li S. Niu H. Hao M. (2013). Critical Role of AKT Protein in Myeloma-Induced Osteoclast Formation and Osteolysis. J. Biol. Chem. 288, 30399–30410. 10.1074/jbc.M113.469973 24005670 PMC3798504

[B4] Chang C.-J. Hung M.-C. (2012). The Role of EZH2 in Tumour Progression. Br. J. Cancer 106, 243–247. 10.1038/bjc.2011.551 22187039 PMC3261672

[B5] Chiang P. K. Burbelo P. D. Brugh S. A. Gordon R. K. Fukuda K. Yamada Y. (1992). Activation of Collagen IV Gene Expression in F9 Teratocarcinoma Cells by 3-deazaadenosine Analogs. Indirect Inhibitors of Methylation. J. Biol. Chem. 267, 4988–4991. 10.1016/s0021-9258(18)42928-6 1537874

[B6] Chiang P. K. Cantoni G. L. (1979). Perturbation of Biochemical Transmethylations by 3-deazaadenosine *In Vivo* . Biochem. Pharmacol. 28, 1897–1902. 10.1016/0006-2952(79)90642-7 454462

[B7] Choudhury S. R. Balasubramanian S. Chew Y. C. Han B. Marquez V. E. Eckert R. L. (2011). (-)-Epigallocatechin-3-gallate and DZNep Reduce Polycomb Protein Level via a Proteasome-dependent Mechanism in Skin Cancer Cells. Carcinogenesis 32, 1525–1532. 10.1093/carcin/bgr171 21798853 PMC3179425

[B8] Coleman R. E. Brown J. Holen I. (2020). “Bone Metastases,” in Abeloff’s Clinical Oncology. Sixth Edition. Editors. Niederhuber J. E. Armitage J. O. Kastan M. B. Doroshow J. H. Tepper J. E. (Philadelphia: Elsevier), 809–830.e3. 10.1016/B978-0-323-47674-4.00056-6

[B9] Costa R. L. B. Han H. S. Gradishar W. J. (2018). Targeting the PI3K/AKT/mTOR Pathway in Triple-Negative Breast Cancer: a Review. Breast Cancer Res. Treat. 169, 397–406. 10.1007/s10549-018-4697-y 29417298

[B10] Escudier B. Powles T. Motzer R. J. Olencki T. Arén Frontera O. Oudard S. (2018). Cabozantinib, a New Standard of Care for Patients with Advanced Renal Cell Carcinoma and Bone Metastases? Subgroup Analysis of the METEOR Trial. J. Clin. Oncol. 36, 765–772. 10.1200/JCO.2017.74.7352 29309249 PMC6804840

[B11] Fiskus W. Wang Y. Sreekumar A. Buckley K. M. Shi H. Jillella A. (2009). Combined Epigenetic Therapy with the Histone Methyltransferase EZH2 Inhibitor 3-deazaneplanocin A and the Histone Deacetylase Inhibitor Panobinostat against Human AML Cells. Blood 114, 2733–2743. 10.1182/blood-2009-03-213496 19638619 PMC2756128

[B12] Gall Trošelj K. Novak Kujundzic R. Ugarkovic D. (2016). Polycomb Repressive Complex’s Evolutionary Conserved Function: the Role of EZH2 Status and Cellular Background. Clin. Epigenetics 8, 1–10. 10.1186/s13148-016-0226-1 27239242 PMC4882774

[B13] Girard N. Bazille C. Lhuissier E. Benateau H. Llombart-Bosch A. Boumediene K. (2014). 3-Deazaneplanocin A (DZNep), an Inhibitor of the Histone Methyltransferase EZH2, Induces Apoptosis and Reduces Cell Migration in Chondrosarcoma Cells. PLoS One 9, e98176–10. 10.1371/journal.pone.0098176 24852755 PMC4031152

[B14] Glazer R. I. Hartman K. D. Knode M. C. Richard M. M. Chiang P. K. Tseng C. K. H. (1986a). 3-Deazaneplanocin: A New and Potent Inhibitor of S-Adenosylhomocysteine Hydrolase and its Effects on Human Promyelocytic Leukemia Cell Line HL-60. Biochem. Biophysical Res. Commun. 135, 688–694. 10.1016/0006-291X(86)90048-3 3457563

[B15] Glazer R. I. Knode M. C. Tseng C. K. H. Haines D. R. Marquez V. E. (1986b). 3-deazaneplanocin A: A New Inhibitor of S-Adenosylhomocysteine Synthesis and its Effects in Human colon Carcinoma Cells. Biochem. Pharmacol. 35, 4523–4527. 10.1016/0006-2952(86)90774-4 3790170

[B16] Goker Bagca B. Ozates N. P. Asik A. Caglar H. O. Gunduz C. Biray Avci C. (2020). Temozolomide Treatment Combined with AZD3463 Shows Synergistic Effect in Glioblastoma Cells. Biochem. Biophysical Res. Commun. 533, 1497–1504. 10.1016/j.bbrc.2020.10.058 33109342

[B17] Guerrero-Zotano A. Mayer I. A. Arteaga C. L. (2016). PI3K/AKT/mTOR: Role in Breast Cancer Progression, Drug Resistance, and Treatment. Cancer Metastasis Rev. 35, 515–524. 10.1007/s10555-016-9637-x 27896521

[B18] Hadji P. Coleman R. Gnant M. (2013). Bone Effects of Mammalian Target of Rapamycin (mTOR) Inhibition with Everolimus. Crit. Rev. Oncology/Hematology 87, 101–111. 10.1016/j.critrevonc.2013.05.015 23838481

[B19] Hayden A. Johnson P. W. M. Packham G. Crabb S. J. (2011). S-Adenosylhomocysteine Hydrolase Inhibition by 3-deazaneplanocin A Analogues Induces Anti-cancer Effects in Breast Cancer Cell Lines and Synergy with Both Histone Deacetylase and HER2 Inhibition. Breast Cancer Res. Treat. 127, 109–119. 10.1007/s10549-010-0982-0 20556507

[B20] He J. Liu Z. Zheng Y. Qian J. Li H. Lu Y. (2012). p38 MAPK in Myeloma Cells Regulates Osteoclast and Osteoblast Activity and Induces Bone Destruction. Cancer Res. 72, 6393–6402. 10.1158/0008-5472.CAN-12-2664 23066034 PMC3525770

[B21] Hemming S. Cakouros D. Codrington J. Vandyke K. Arthur A. Zannettino A. (2017). EZH2 Deletion in Early Mesenchyme Compromises Postnatal Bone Microarchitecture and Structural Integrity and Accelerates Remodeling. FASEB j. 31, 1011–1027. 10.1096/fj.201600748R 27934660

[B22] Hortobagyi G. N. (2015). Everolimus Plus Exemestane for the Treatment of Advanced Breast Cancer: A Review of Subanalyses from BOLERO-2. Neoplasia 17, 279–288. 10.1016/j.neo.2015.01.005 25810012 PMC4372651

[B23] Hu G.-F. Wang C. Hu G.-X. Wu G. Zhang C. Zhu W. (2020). AZD3463, an IGF-1R Inhibitor, Suppresses Breast Cancer Metastasis to Bone via Modulation of the PI3K-Akt Pathway. Ann. Transl. Med. 8, 336. 10.21037/atm.2020.02.110 32355780 PMC7186597

[B24] Huang H. Chang E.-J. Ryu J. Lee Z. H. Lee Y. Kim H.-H. (2006). Induction of C-Fos and NFATc1 during RANKL-Stimulated Osteoclast Differentiation Is Mediated by the P38 Signaling Pathway. Biochem. Biophysical Res. Commun. 351, 99–105. 10.1016/j.bbrc.2006.10.011 17052691

[B25] Jensen A. B. Wynne C. Ramirez G. He W. Song Y. Berd Y. (2010). The Cathepsin K Inhibitor Odanacatib Suppresses Bone Resorption in Women with Breast Cancer and Established Bone Metastases: Results of a 4-week, Double-Blind, Randomized, Controlled Trial. Clin. Breast Cancer 10, 452–458. 10.3816/CBC.2010.n.059 21147688

[B26] Kleer C. G. Cao Q. Varambally S. Shen R. Ota I. Tomlins S. A. (2003). EZH2 Is a Marker of Aggressive Breast Cancer and Promotes Neoplastic Transformation of Breast Epithelial Cells. Proc. Natl. Acad. Sci. 100, 11606–11611. 10.1073/pnas.1933744100 14500907 PMC208805

[B27] Kops G. J. P. L. Ruiter N. D. d. De Vries-Smits A. M. M. Powell D. R. Bos J. L. Burgering B. M. T. (1999). Direct Control of the Forkhead Transcription Factor AFX by Protein Kinase B. Nature 398, 630–634. 10.1038/19328 10217147

[B28] Lee R. J. Smith M. R. (2013). Targeting MET and Vascular Endothelial Growth Factor Receptor Signaling in Castration-Resistant Prostate Cancer. Cancer J. (United States 19, 90–98. 10.1097/PPO.0b013e318281e280 PMC3683553 23337762

[B29] Li X. Udagawa N. Itoh K. Suda K. Murase Y. Nishihara T. (2002). p38 MAPK-Mediated Signals Are Required for Inducing Osteoclast Differentiation but Not for Osteoclast Function. Endocrinology 143, 3105–3113. 10.1210/endo.143.8.8954 12130576

[B30] Liang W. Wang F. Chen Q. Dai J. Escara-Wilke J. Keller E. T. (2019). Targeting Cathepsin K Diminishes Prostate Cancer Establishment and Growth in Murine Bone. J. Cancer Res. Clin. Oncol. 145, 1999–2012. 10.1007/s00432-019-02950-y 31172267 PMC6658578

[B31] Liu S. Wolfe M. S. Borchardt R. T. (1992). Rational Approaches to the Design of Antiviral Agents Based on S-Adenosyl-L-Homocysteine Hydrolase as a Molecular Target. Antiviral Res. 19, 247–265. 10.1016/0166-3542(92)90083-H 1444329

[B32] Marie P. J. (2012). Signaling Pathways Affecting Skeletal Health. Curr. Osteoporos. Rep. 10, 190–198. 10.1007/s11914-012-0109-0 22711369

[B33] Moharram S. A. Shah K. Khanum F. Rönnstrand L. Kazi J. U. (2019). The ALK Inhibitor AZD3463 Effectively Inhibits Growth of Sorafenib-Resistant Acute Myeloid Leukemia. Blood Cancer J. 9, 1–4. 10.1038/s41408-018-0169-1 PMC6333797 30647405

[B34] Moon J. B. Kim J. H. Kim K. Youn B. U. Ko A. Lee S. Y. (2012). Akt Induces Osteoclast Differentiation through Regulating the GSK3β/NFATc1 Signaling Cascade. J.I. 188, 163–169. 10.4049/jimmunol.1101254 22131333

[B35] Nagel S. Venturini L. Marquez V. E. Meyer C. Kaufmann M. Scherr M. (2010). Polycomb Repressor Complex 2 Regulates HOXA9 and HOXA10, Activating ID2 in NK/T-cell Lines. Mol. Cancer 9, 151–212. 10.1186/1476-4598-9-151 20565746 PMC2894765

[B36] Ozates N. P. Soğutlu F. Lerminoglu F. Demir B. Gunduz C. Shademan B. (2021). Effects of Rapamycin and AZD3463 Combination on Apoptosis, Autophagy, and Cell Cycle for Resistance Control in Breast Cancer. Life Sci. 264, 118643. 10.1016/j.lfs.2020.118643 33141044

[B55] Pang Y. Fu Y. Li C. Wu Z. Cao W. Hu X. (2020). Metal-Organic Framework Nanoparticles for Ameliorating Breast Cancer-Associated Osteolysis. Nano Lett. 20, 829–840. 10.1021/acs.nanolett.9b02916 31916446

[B37] Pérez-Tenorio G. Stål O. Stål O. Malmström A. Nordenskjöld B. Nordenskjöld K. (2002). Activation of Akt/PKB in Breast Cancer Predicts a Worse Outcome Among Endocrine Treated Patients. Br. J. Cancer 86, 540–545. 10.1038/sj.bjc.6600126 11870534 PMC2375266

[B38] Piunti A. Pasini D. (2011). Epigenetic Factors in Cancer Development: Polycomb Group Proteins. Future Oncol. 7, 57–75. 10.2217/fon.10.157 21174538

[B39] Puppe J. Drost R. Liu X. Joosse S. A. Evers B. Cornelissen-Steijger P. (2009). BRCA1-deficient Mammary Tumor Cells Are Dependent on EZH2 Expression and Sensitive to Polycomb Repressive Complex 2-inhibitor 3-deazaneplanocin A. Breast Cancer Res. 11, 1–12. 10.1186/bcr2354 PMC2750125 19709408

[B40] Sampson V. B. Vetter N. S. Kamara D. F. Collier A. B. Gresh R. C. Kolb E. A. (2015). Vorinostat Enhances Cytotoxicity of SN-38 and Temozolomide in ewing Sarcoma Cells and Activates STAT3/AKT/MAPK Pathways. PLoS One 10, e0142704–19. 10.1371/journal.pone.0142704 26571493 PMC4646493

[B41] Smith D. C. Smith M. R. Sweeney C. Elfiky A. A. Logothetis C. Corn P. G. (2013). Cabozantinib in Patients with Advanced Prostate Cancer: Results of a Phase II Randomized Discontinuation Trial. J. Clin. Oncol. 31, 412–419. 10.1200/JCO.2012.45.0494 23169517 PMC4110249

[B42] Smith M. De Bono J. Sternberg C. Le Moulec S. Oudard S. De Giorgi U. (2016). Phase III Study of Cabozantinib in Previously Treated Metastatic Castration-Resistant Prostate Cancer: COMET-1. J. Clin. Oncol. 34, 3005–3013. 10.1200/JCO.2015.65.5597 27400947

[B43] Sousa S. Clézardin P. (2018). Bone-Targeted Therapies in Cancer-Induced Bone Disease. Calcif. Tissue Int. 102, 227–250. 10.1007/s00223-017-0353-5 29079995

[B44] Stitt T. N. Drujan D. Clarke B. A. Panaro F. Timofeyva Y. Kline W. O. (2004). The IGF-1/PI3K/Akt Pathway Prevents Expression of Muscle Atrophy-Induced Ubiquitin Ligases by Inhibiting FOXO Transcription Factors. Mol. Cel 14, 395–403. 10.1016/S1097-2765(04)00211-4 15125842

[B45] Sugatani T. Hruska K. A. (2005). Akt1/Akt2 and Mammalian Target of rapamycin/Bim Play Critical Roles in Osteoclast Differentiation and Survival, Respectively, whereas Akt Is Dispensable for Cell Survival in Isolated Osteoclast Precursors. J. Biol. Chem. 280, 3583–3589. 10.1074/jbc.M410480200 15545269

[B46] Suvà M.-L. Riggi N. Janiszewska M. Radovanovic I. Provero P. Stehle J.-C. (2009). EZH2 Is Essential for Glioblastoma Cancer Stem Cell Maintenance. Cancer Res. 69, 9211–9218. 10.1158/0008-5472.CAN-09-1622 19934320

[B47] Tan J. Yang X. Zhuang L. Jiang X. Chen W. Lee P. L. (2007). Pharmacologic Disruption of Polycomb-Repressive Complex 2-mediated Gene Repression Selectively Induces Apoptosis in Cancer Cells. Genes Dev. 21, 1050–1063. 10.1101/gad.1524107 17437993 PMC1855231

[B48] Uehara S. Udagawa N. Kobayashi Y. (2019). Regulation of Osteoclast Function via Rho-Pkn3-C-Src Pathways. J. Oral Biosci. 61, 135–140. 10.1016/j.job.2019.07.002 31400545

[B49] Wang Y. Wang L. Guan S. Cao W. Wang H. Chen Z. (2016). Novel ALK Inhibitor AZD3463 Inhibits Neuroblastoma Growth by Overcoming Crizotinib Resistance and Inducing Apoptosis. Sci. Rep. 6, 1–10. 10.1038/srep19423 26786851 PMC4726162

[B50] Xie Z. Bi C. Cheong L. L. Liu S. C. Huang G. Zhou J. (2011). Determinants of Sensitivity to DZNep Induced Apoptosis in Multiple Myeloma Cells. PLoS One 6, e21583. 10.1371/journal.pone.0021583 21720561 PMC3123372

[B51] Yoo K. H. Hennighausen L. (2012). EZH2 Methyltransferase and H3K27 Methylation in Breast Cancer. Int. J. Biol. Sci. 8, 59–65. 10.7150/ijbs.8.59 22211105 PMC3226033

[B52] Zheng S. Xiao L. Liu Y. Wang Y. Cheng L. Zhang J. (2018). DZNep Inhibits H3K27me3 Deposition and Delays Retinal Degeneration in the Rd1 Mice. Cell Death Dis. 9, 1–14. 10.1038/s41419-018-0349-8 29472543 PMC5833420

[B53] Zheng X.-j. Li W. Yi J. Liu J.-Y. Ren L. W. Zhu X.-M. (2020). EZH2 Regulates Expression of FOXC1 by Mediating H3K27me3 in Breast Cancers. Acta Pharmacol. Sin. 0, 1–9. 10.1038/s41401-020-00543-x PMC8209002 33057161

