# Supplementary figures and images for: Combination of AZD3463 and DZNep Prevents Bone Metastasis of Breast Cancer by Suppressing Akt Signaling
Source: Front Pharmacol. 2021 May 28;12:652071. doi: 10.3389/fphar.2021.652071 (PMC8193724; doi:10.3389/fphar.2021.652071)

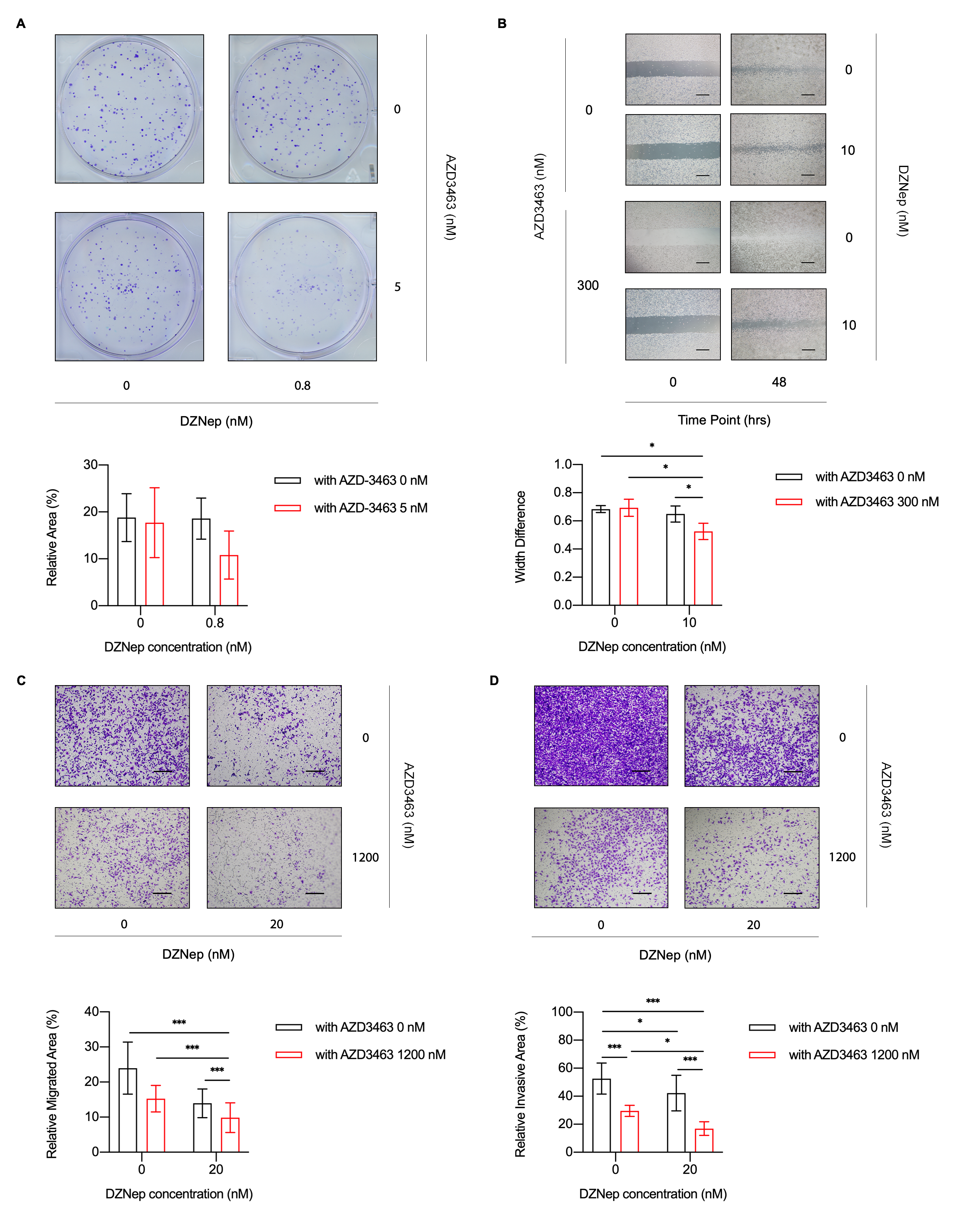

Supplement: Supplementary file 1 [file Image3.TIF]

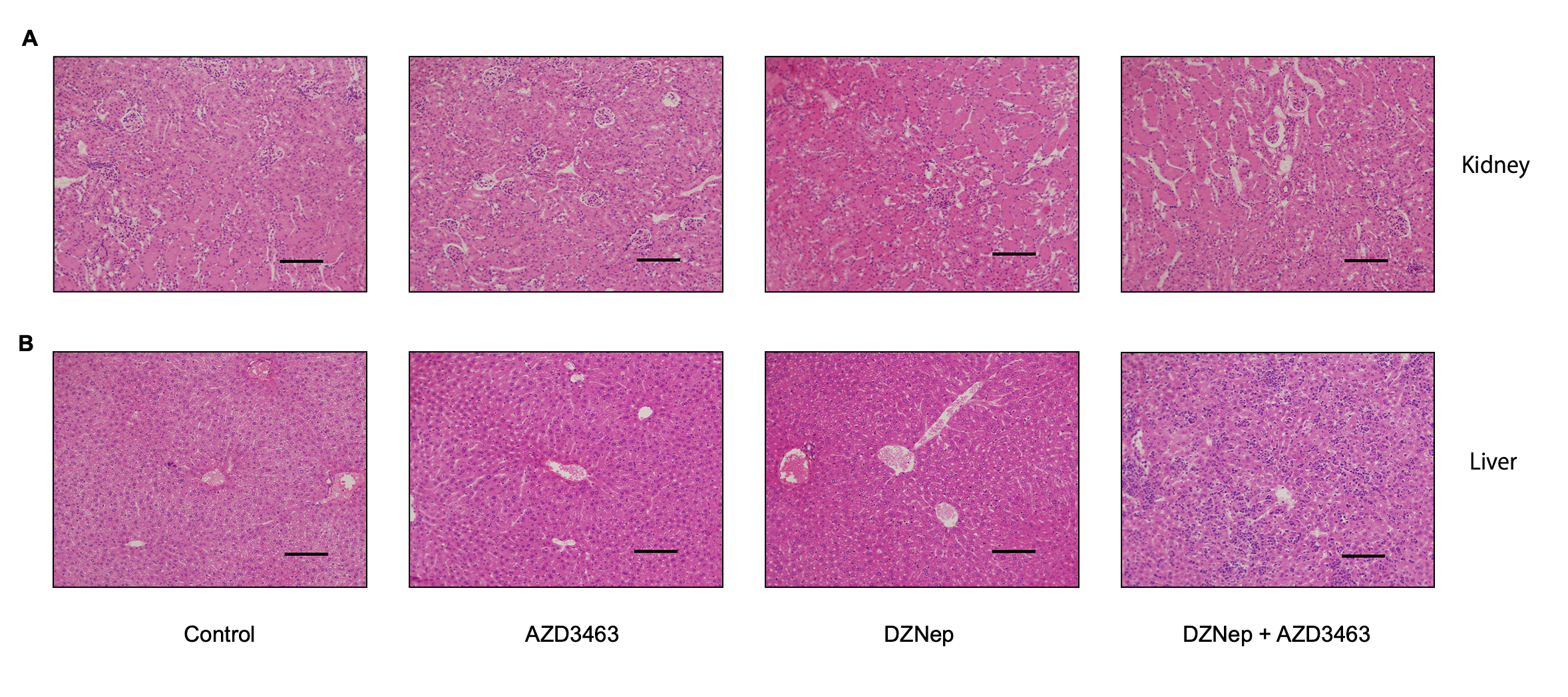

Supplement: Supplementary file 2 [file Image4.TIF]

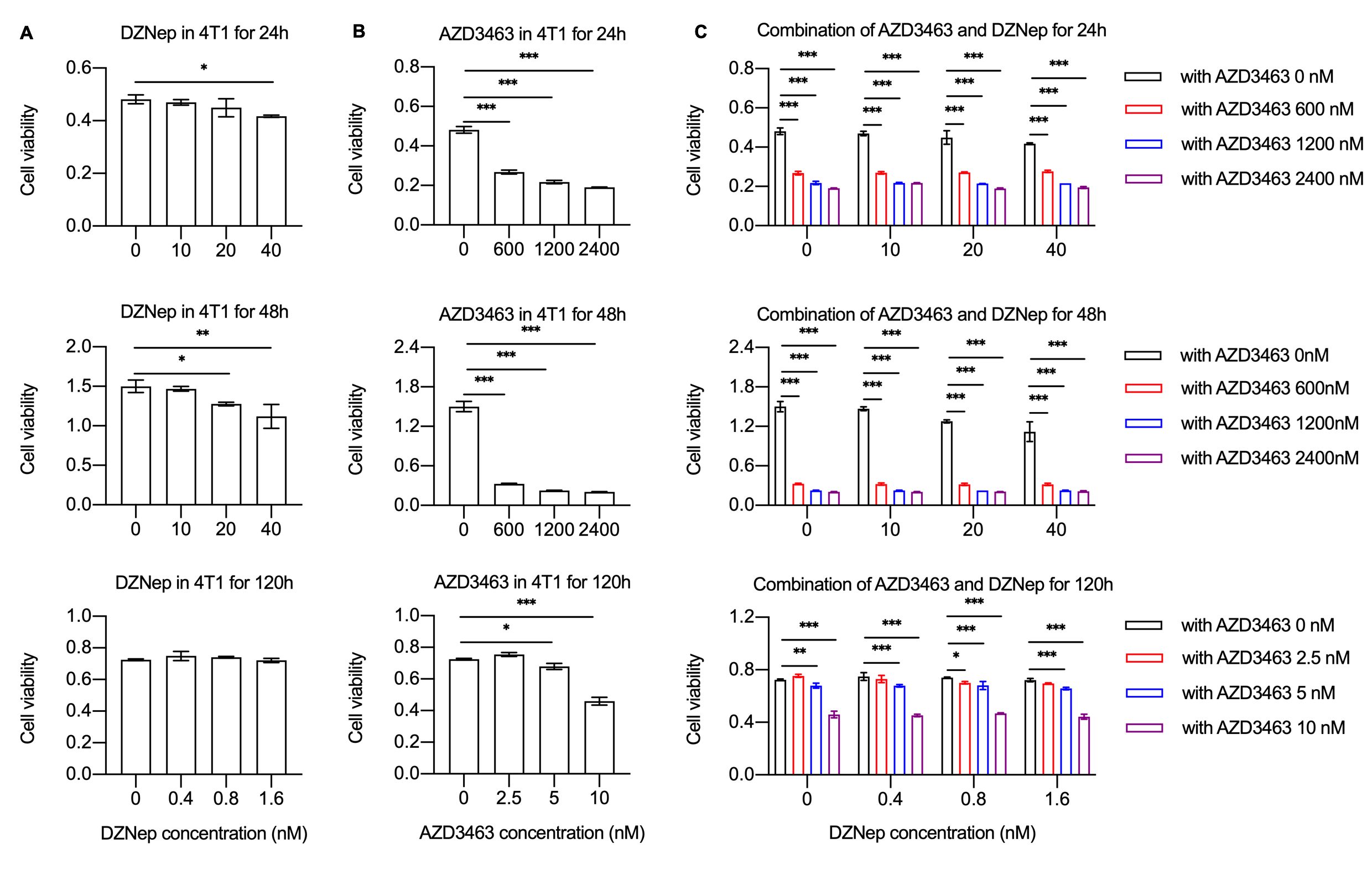

Supplement: Supplementary file 3 [file Image2.TIF]

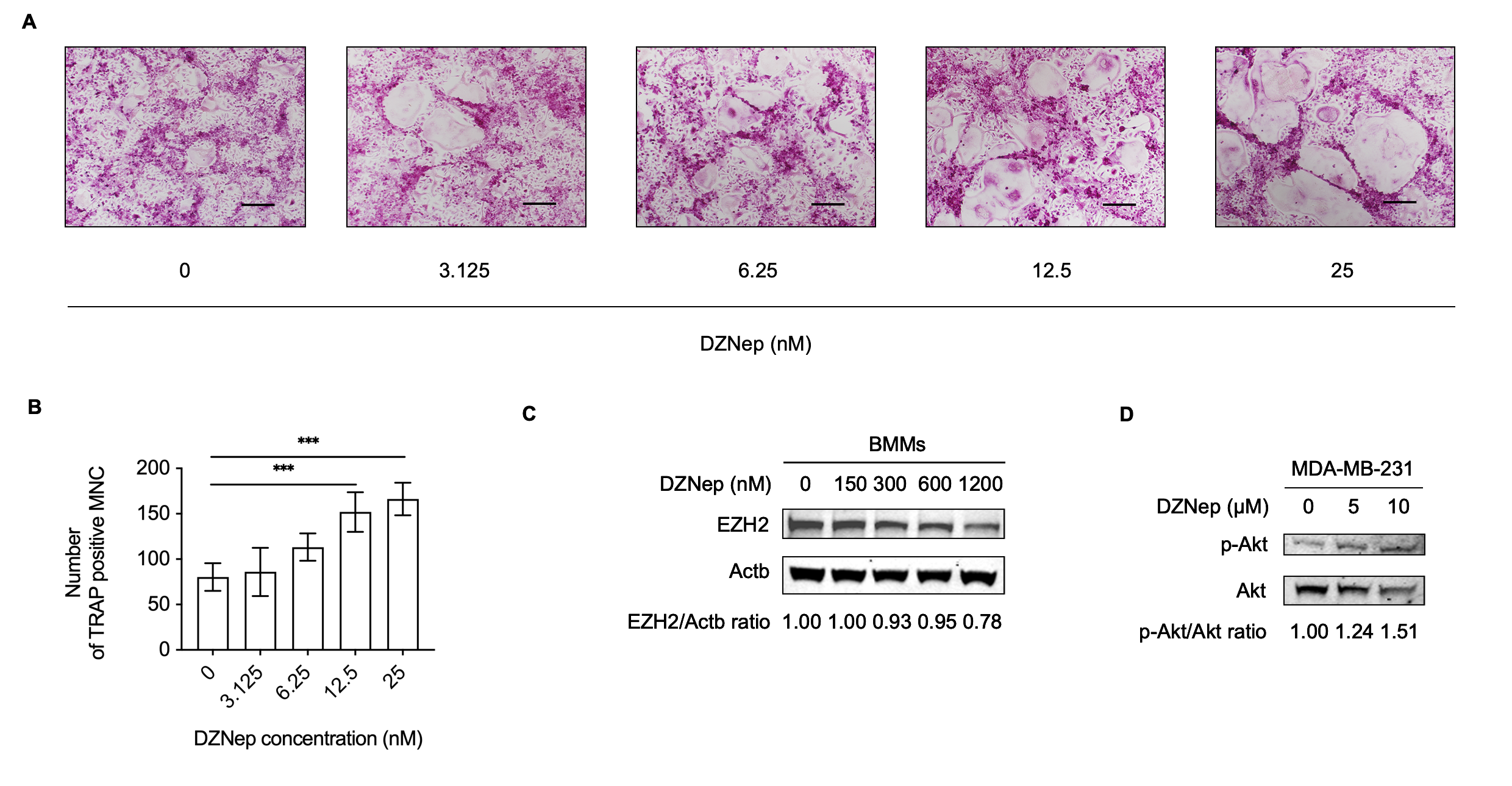

Supplement: Supplementary file 4 [file Image1.TIF]
